# Indian summer monsoon variability forecasts in the North American multimodel ensemble

**DOI:** 10.1007/s00382-018-4203-6

**Published:** 2018-04-12

**Authors:** Bohar Singh, Ben Cash, James L. Kinter III

**Affiliations:** 1 George Mason University, Fairfax, VA 22031 USA; 2 Center for Ocean-Land-Atmosphere Studies, George Mason University, Fairfax, VA 22031 USA

**Keywords:** Indian Monsoon, Interannual variability, Teleconnections

## Abstract

The representation of the seasonal mean and interannual variability of the Indian summer monsoon rainfall (ISMR) in nine global ocean-atmosphere coupled models that participated in the North American Multimodal Ensemble (NMME) phase 1 (NMME:1), and in nine global ocean-atmosphere coupled models participating in the NMME phase 2 (NMME:2) from 1982–2009, is evaluated over the Indo-Pacific domain with May initial conditions. The multi-model ensemble (MME) represents the Indian monsoon rainfall with modest skill and systematic biases. There is no significant improvement in the seasonal forecast skill or interannual variability of ISMR in NMME:2 as compared to NMME:1. The NMME skillfully predicts seasonal mean sea surface temperature (SST) and some of the teleconnections with seasonal mean rainfall. However, the SST-rainfall teleconnections are stronger in the NMME than observed. The NMME is not able to capture the extremes of seasonal mean rainfall and the simulated Indian Ocean-monsoon teleconnections are opposite to what are observed.

## Introduction

The variations of the seasonal rainfall associated with the south Asian monsoon are enormously important for millions of lives on the Indian subcontinent and beyond. The spatial and temporal variations of rainfall have a significant impact on the agrarian economies of India, Bangladesh and Pakistan. While interannual variations in Indian summer monsoon rainfall (ISMR) are only $\approx$ 10% of the long term mean, the high and low extremes of the seasonal mean ISMR result in floods and droughts (Shukla and Moolay 1987). Food production in the Indian region is strongly correlated with ISMR (Gadgil et al. 1999), and these floods and droughts can cause devastating human and economic losses. The south Asian monsoon is recognized as a prominent feature of the global circulation (Lau and K.-M. Kim 2006). Continental-scale land-sea contrast has been suggested as primary cause for the monsoon (Webster et al. 1998), while other studies suggest it is driven by the meridional movement of the Intra-Tropical Convergence Zone (ITCZ) (Gadgil et al. 2003). Besides these two basic components the ISMR is also influenced by the topography of Great Himalaya, which introduces an elevated heating source and helps to set the meridional tropospheric temperature gradient. The local reversal of the meridional tropospheric temperature gradient during the summer is thought to be important for the onset of the ISMR. This gradient is maintained in part by the heat fluxes and diabatic heating due to precipitation (Yanai et al. 1992; Wu and Zhang 1998). The topography of Himalaya isolates the Indian monsoon thermal maximum from the dry and cold air in the interior of Asian continent (Chakraborty et al. 2002; Boos and Kuang 2010), and numerical modeling studies have found that by removing the topography the northern extent of the precipitation is greatly reduced (e.g., Hahn and Manabe 1975; Prell and Kutzbach 1992). Another key feature of the monsoon circulation is the climatological low over northwestern India and Pakistan, which is the deepest low in the global tropics during boreal summer (Joshi and Desai 1985; Sikka 1997). It develops in April–May concurrently with the south-westerly wind regime (Ramage 1996). The high winds associated with the monsoon trough not only bring moisture over the land but also natural dust and aerosols. Aerosols can influence the monsoon through direct (interaction with solar radiation) and indirect (interaction with cloud microphysics) effects (Bollasina et al. 2011; Lau and K.-M. Kim 2006). Slowly varying boundary conditions such as SST, snow cover and soil moisture are also key components of the Indian monsoon, particularly in terms of its potential predictability (Charney and Shukla 1981). The teleconnection between southern oscillation and ISMR is among the oldest observed teleconnections (Walker 1925). Observational analysis shows that indian summer monsoon rainfall found below average during El Niño events, while La Niña events lead to above normal rainfall (e.g. Sikka 1980; Pant and Parthasarathy 1981; Rasmusson and Carpenter 1983; Gadgil et al. 2003, 2004). Niño 3.4 index (standardized area average SST average over the region 170$^{\circ }$E–120$^{\circ }$W, 5$^{\circ }$S–5$^{\circ }$N) is negatively correlated with ISMR. The observed negative correlation between the ISMR and Niño 3.4 index can be explained to some extent by the modulation of the Walker circulation (Shukla and Paolino 1983; Palmer et al. 1992). Thus, the Indian monsoon includes a complex orographically influenced structure, interaction between convection and large-scale atmospheric circulation, wave propagation in both the zonal and meridional directions, air-sea interaction, and cloud-aerosol interaction. Due to the presence of all the above components and their nonlinear interactions, Indian monsoon rainfall is an extremely challenging phenomenon to simulate (Gadgil et al. 2005).

Uncertainties and model errors in climate prediction can be classified into two groups: (1) uncertainties and errors in model initialization and (2) uncertainties and errors in model parameterizations and model physics (Buizza et al. 2005; Schwierz et al. 2006). The multi-model ensemble (MME) is recognized as one approach to address the above-mentioned uncertainties and errors (Palmer et al. 2004, 2005; Hagedorn et al. 2004). MMEs typically have higher skill for predicting weather and climate as compared to single models, and also provide estimates of model uncertainty. The simulation and prediction of ISMR at both inter-annual and intra-seasonal time scales has been evaluated in several such MMEs (Gadgil and Sajani 1998; Kang et al. 2002; Rajeevan and Nanjundiah 2009; Sperber et al. 2013; Wang et al. 2004, 2004). All MMEs examined previously have been shown to simulate large-scale feature of Indian rainfall with modest skill. Some studies (Wang et al. 2003; Sharmila et al. 2013) have highlighted the importance of air-sea interactions and suggest that coupled ocean-atmospheric models are crucial for monsoon seasonal predictions. Preethi et al. (2010) and Rajeevan et al. (2012) evaluated the seasonal forecast skills of Development of European multi-model ensemble system for seasonal to interannual predictions (DEMETER) (Palmer et al. 2004) and ENSEMBLE (Hewitt and Griggs 2004) projects respectively and found that these multi-model ensembles predict ISMR with positive (modest) skill. The realized skill is still below the limit of potential predictability (Saha et al. 2016).

In this study we investigate the ability of the North-American Multi Model Ensemble (NMME) models to reproduce and predict the seasonal mean and interannual variability of the Indian summer monsoon rainfall. The NMME is a collaborative effort between several modeling centers for seasonal forecasts. The NMME simulations provides us with the opportunitiy to compare the simulations from multiple seasonal models for the same phenomenon. The analysis of the multi-model simulations for identical scenarios will aid us in identifying and understanding the similarities and differences of the various model simulations. The study of Kirtman et al. (2014) have shown that modeling system improvements and data assimilation system improvements led to improved NMME-2 forecast quality. The second objective of this study is to compare the seasonal forecast skill of NMME phase 1 with the currently operational NMME phase 2 to understand whether the improvements in modeling systems and data assimilation systems have contributed to improved seasonal prediction of the Indian summer monsoon.

## Data and methodology

The NMME is an MME producing both retrospective and real-time intraseasonal to interannual predictions and is comprised of global coupled atmosphere-ocean models from modeling centers in the United States and Canada (Kirtman et al. 2014). The NMME provides retrospective seasonal forecasts for 1982–2010. In this study nine models are selected from the first implementation of the NMME (phase 1; denoted here as NMME:1) and nine models from the current implementation (phase 2; denoted here as NMME:2 as summarized in Table 1. CFSv2, CanCM3 and CanCM4 are the common models in both of the NMME phases (denoted by $\oplus$ in Table 1). The 15 models have a common re-forecast period of 28 years from 1982–2009. The number of ensemble members for each model ranges from 6 to 24, with 109 total ensemble members from nine models for NMME:1, and 110 ensemble members from nine participating models for NMME:2. Model runs are initialized every month with forecast lengths ranging from 6 to 11 months. In the present study we analyze the June–September (JJAS) seasonal means of precipitation and SST for forecasts starting from May 1 initial conditions. Equal weights are given to each model in calculating the average over all models and ensemble members, denoted the multimodel ensemble mean (MMEM). The choice of reforecasts initialized in May was made in order to avoid inclusion of potential skill from the atmospheric initial conditions. It is assumed that after one month of model integration, the atmospheric initial conditions, which provide much of the skill for numerical weather forecasts at 1–15 days lead-time, have a minimal impact on the forecast skill of the ensuing seasonal mean. It is possible that forecasts initialized in May are subject to the spring predictability barrier (Torrence and Webster 1998), which may mask some of the difference in skill among models. All NMME models are re-gridded to a common 1$^{\circ } \times 1^{\circ }$ resolution. The Climate Prediction Center Merged Analysis of Precipitation (CMAP) (Xie and Arkin 1997) and the optimum interpolation version 2 analysis of Reynolds et al. (2002) (OISSTv2) dataset at 1$^{\circ } \times 1^{\circ }$ resolution are used as the observed precipitation and sea surface temperature, respectively. It should be noted that previous work (Cash et al. 2008) has hown that satellite based observational rainfall data products, CMAP and Global Precipitation Climatology Project (GPCP; Huffman et al. 1997) have some significant differences over the ISMR region. Rainfall maxima over Bay of Bengal has high magnitude in the CMAP as compared to GPCP. Rainfall maxima over Western Ghats is also found higher in CMAP as compared to GPCP. Therefore some of the conclusions of this study may be sensitive to the choice of CMAP as the observed rainfall product.

**Table 1:** List of NMME models used for this study

Model	Hindcast period	Ensemble size	Lead time (months)	Model res. (Atoms)	Model res. (ocean)	References
Operational NMME models						
NCEP/$CFSv2^{\oplus }$	1982–2010	24	0–9	T126L64	MOM4L40 $0.25^{\circ }$ Eq	Saha et al. (2014)
GFDL/CM2p1 aer04	1982–2010	10	0–11	$2\times 2.5^{\circ }$L24	MOM4L50 $0.3^{\circ }$ Eq	Delworth et al. (2006)
GFDL/CM2p5 FLORB01	1982–2010	12	0–11	C18L32 (50 Km)	MOM5L50 $0.3^{\circ }$ Eq $1^{\circ }$Polar1.5	Vecchi et al. (2014)
GFDL/CM2p5 FLORA06	1982–2010	12	0–11	C18L32 (50 Km)	MOM5L50 $0.3^{\circ }$ Eq $1^{\circ }$Polar1.5	Vecchi et al. (2014)
Can$CM3^{\oplus }$	1982–2010	10	0–11	CanAM3 T63L31	CanOM4L40 $0.94^{\circ }$ Eq	Merryfieldet al. (2013)
Can$CM4^{\oplus }$	1982–2010	10	0–11	CanAM4 T63L35	CanOM4L40 $0.94^{\circ }$ Eq	Merryfieldet al. (2013)
NCAR/CCSM4	1982–2010	10	0–11	$0.9\times 1.25^{\circ }$L26	POPL60 $0.25^{\circ }$ Eq	Infanti and Kirtman (2016)
NASA/GMAO 062012	1982–2010	11	0–11	$1\times 1.25^{\circ }$L72	MOM4L40 $0.25^{\circ }$ Eq	Vernieres et al. (2012)
NCAR/CESM1	1982–2010	10	0–11	$0.9\times 1.25^{\circ }$L30	POPL60 $0.25^{\circ }$ Eq	Tribbia (2015)
Retired or old versions of the NMME models						
NCEP/CFSv1	1982–2009	15	0–8	T62L64	MOM3L40 $0.3^{\circ }$ Eq	Saha et al. (2006)
NCAR/CCSM3	1982–2010	6	0–11	T85L26	POPL42 $0.3^{\circ }$ Eq	Kirtman and Min (2009)
NASA/GMAO	1982–2010	11	0–11	$1\times 1.25^{\circ }$L72	MOM4L40 $0.25^{\circ }$ Eq	Vernieres et al. (2012)
IRI/ECHAM4f	1982–2010	12	0–7	T42L19	MOM3L25 $1.5^{\circ }\times 0.5^{\circ }$	DeWitt (2005)
IRI/ECHAM4a	1982–2010	12	0–7	T42L19	MOM3L25 $1.5^{\circ }\times 0.5^{\circ }$	DeWitt (2005)
GFDL/CM2p1	1982–2010	10	0–11	$2\times 2.5^{\circ }$L24	MOM4L50 $0.3^{\circ }$ Eq	Delworth et al. (2006)

### Results and discussions

**Fig. 1:**
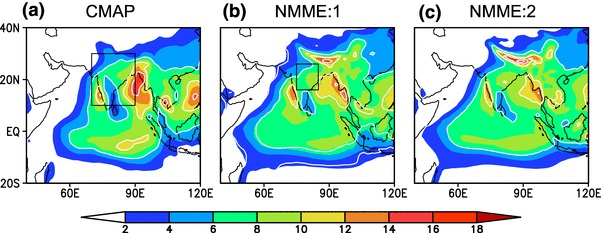
Spatial distribution of JJAS mean rainfall (mm/day) over the period of 1982–2009, **a** from CMAP, **b** from MMEM of NMME:1, **c** from MMEM of NMME:2, rectangle over figure **a** represents extend monsoon region (70$^{\circ }$E–90$^{\circ }$E, 10$^{\circ }$N–30$^{\circ }$N),rectangle over figure **b** represents Central India region (75$^{\circ }$E–85$^{\circ }$E, 16$^{\circ }$N–26$^{\circ }$N). All the forecasts are initialized in May and verified for JJAS mean

The JJAS mean precipitation from CMAP and the multi model ensemble mean (MMEM) of NMME:1 and NMME:2 are shown in Fig. (1a–c). Local rainfall maxima over the Western Ghats, Bay of Bengal and the south equatorial ocean are well simulated in both NMME:1 and NMME:2, while rainfall over the foothills of the Himalaya is overestimated in both phases. Spatial correlations of seasonal mean rainfall over the Indo-Pacific domain are 0.92 and 0.89 for NMME:1 and NMME:2, respectively. Seasonal mean rainfall over the region outlined in Fig. 1a (70$^{\circ }$E–90$^{\circ }$E, 10$^{\circ }$N–30$^{\circ }$N) is 7.25 mm/day for the observations, while it is 7.93 mm/day and 7.73 mm/day for NMME:1 and NMME:2, respectively.

**Fig. 2:**
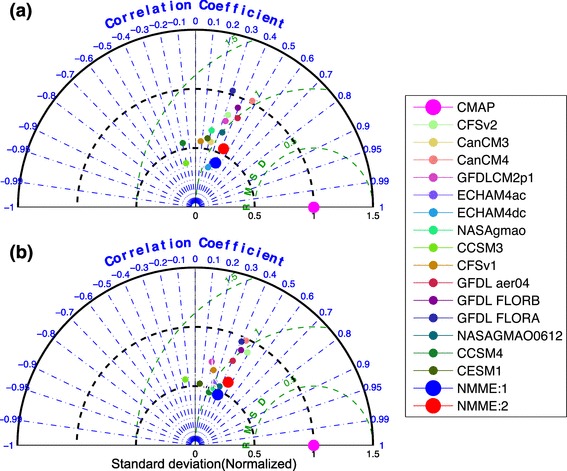
Taylor diagram area averaged seasonal mean rainfall, **a** over central India (75$^{\circ }$E–85$^{\circ }$E, 16$^{\circ }$N–26$^{\circ }$N, Ocean data points are excluded), **b** over the region selected in figure **1a**: (70$^{\circ }$E–90$^{\circ }$E, 10$^{\circ }$N–30$^{\circ }$N) for individual NMME models and NMME:1 and NMME:2 as compared to Observations (CMAP) over the period of 1982–2009. All the forecasts are initialized in May and verified for JJAS mean

**Fig. 3:**
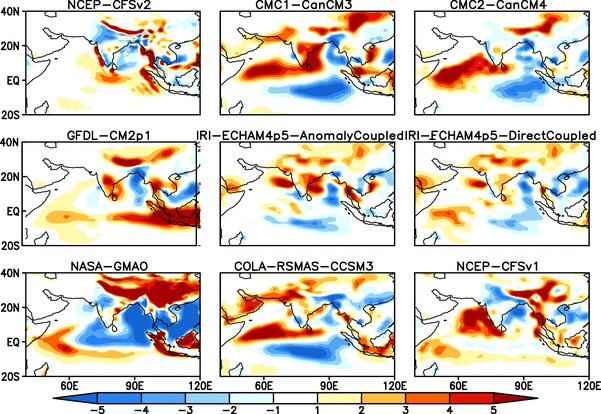
Difference (Model—Obs.) between ensemble mean JJAS climatological rainfall of NMME:1 and Observations (CMAP) from 1982–2009, all the forecasts are initialized in May and verified for JJAS mean

**Fig. 4:**
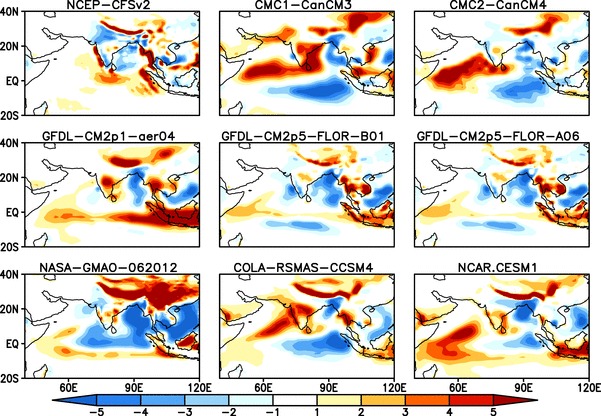
Difference (Model—Obs.) between ensemble mean JJAS climatological rainfall of NMME:2 and Observations (CMAP) from 1982–2009, all the forecasts are initialized in May and verified for JJAS mean

In general, Fig. 1 indicates that the seasonal mean monsoon rainfall is well represented in the NMME but that there is no significant improvement in NMME:2 as compared to NMME:1. This is further illustrated by considering the Taylor diagram (Fig. 2) of area averaged seasonal mean rainfall from the ensemble mean of the NMME models and CMAP data over Central India (CI: 75$^{\circ }$E-85$^{\circ }$E,16$^{\circ }$N-26$^{\circ }$N; box shown over Fig. 1b) and over a larger region (70$^{\circ }$E-90$^{\circ }$E, 10$^{\circ }$N-30$^{\circ }$N; box shown over Fig. 1a) that includes the western Ghats and part of the Bay of Bengal. Most of the NMME models are closely clustered on Taylor diagram around 0.2–0.5, with the exception of CCSM3 and CCSM4 which are negatively correlated with observations. It is again hard to distinguish between the NMME:1 and NMME:2 models in this metric, emphasizing that there is no significant improvement seasonal forecast skill in NMME:2 relative to NMME:1. The ensemble mean of each NMME model consistently has less year-to-year variability as compared to observations for the selected regions. The MMEMs for each phase do show some differences in skill, predicting seasonal mean rainfall over India with moderate correlations of 0.4 and 0.5 in NMME:1 and NMME:2, respectively. The reduced interannual variability shown by the model ensemble means (Fig. 2a, b) is due to the averaging of individual members, which increases the signal-to-noise ratio. We have examined the interannual variability of the individual model realizations (results not shown) and found that the interannual variability of the individual model realizations is comparable to the observations. Interannual variability of the MMEM of NMME:2 is larger than that of NMME:1, but the difference is statistically insignificant at the 95% confidence level.

The differences between simulated and observed climatological seasonal mean rainfall for the NMME:1 and NMME:2 models are shown in Figs. 3 and 4, respectively. There are several intramodel differences and similarities among the NMME seasonal rainfall simulations. Almost all of the models overestimate rainfall over the foothills of the Himalayas, the western and equatorial Indian Ocean, and underestimate rainfall over the Bay of Bengal. Over the Indian land region ECHAM4p5-AC, ECHAM4p5-DC, CCSM3, Can-CM3 and CCSM4 overestimate rainfall, while both versions of CFS and NASA-GMAO underestimate rainfall. Can-CM4 and GFDL-CM2p5-FLOR simulate seasonal mean rainfall reasonably well.

**Fig. 5:**
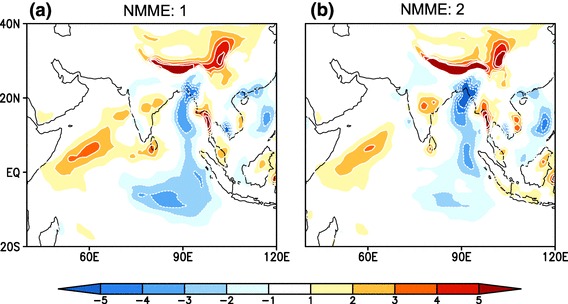
Difference between multimodel ensemble mean JJAS climatological rainfall from observations (NMME - Obs.), **a** for NMME:1 and **b** for NMME:2, over the period of 1982–2009

When we consider the MMEMs for NMME:1 and NMME:2 (Fig. 5) the large intramodel difference are mostly canceled out and the root mean square error (RMSE) over the study region (see Fig. 1) is 2.76 and 2.29 mm/day for NMME:1 and NMME:2, respectively. Rainfall over the foothills of Himalaya is overestimated in both MMEMs by more than 5 mm/day. Both MMEMs also overestimate the seasonal mean rainfall over the western Indian Ocean and underestimate it over the Bay of Bengal, although the wet bias over western Indian ocean and dry bias over Bay of Bengal are reduced in NMME:2. Over the land mass region of India rainfall is well simulated in both of phases of NMME. The ensemble means of the seasonal mean ISMR was also analyzed separately for the two phases, after excluding common models (CFSv2, CMC1 and CMC2) and the conclusions are remain same (figures not shown). Taken together we find there is no significant improvement in seasonal mean rainfall over the Indo-Pacific domain from NMME:1 and NMME:2, despite the inclusion of improved versions of the participating models in NMME:2.We have analyzed the ensemble mean of seasonal mean rainfall from older version models (NASA-GMAO, NCAR-CCSM3 and GFDLCM2p1) and improved version of similar models (NASA-GMAO, NCAR-CCSM4 and GFDLCM2p5-FLOR) and found that improvement in each modeling system is small as compared to their individual mean bias (figures are not shown). Therefore the improvements in modeling systems are not being reflected in the simulations of the seasonal mean ISMR.

**Fig. 6:**
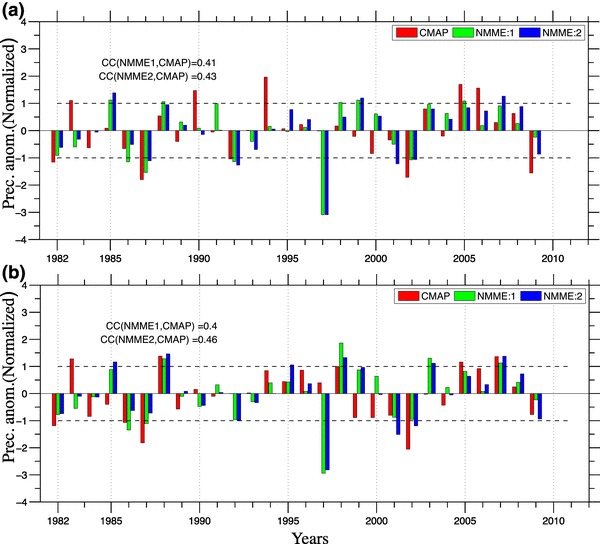
Histograms of standardized seasonal mean monsoon rainfall anomaly averaged over **a** over CI: (75$^{\circ }$E–85$^{\circ }$E, 16$^{\circ }$N–26$^{\circ }$N, Ocean data points are excluded), **b** over the region selected in figure **1a**: (70$^{\circ }$E–90$^{\circ }$E, 10$^{\circ }$N–30$^{\circ }$N), form Observations (red bars), multimodel ensemble mean NMME:1 (green bars) and multimodel ensemble mean NMME:2 (blue bars) for the period 1982–2009, dashed black line denotes ±1 standard deviation of seasonal anomalies, abbreviation CC is stands for correlation coefficient

**Table 2:** Heidke skill score for forecast verification

Phase	Above normal	Normal	Below normal
CI: (75$^{\circ }$E–85$^{\circ }$E, 16$^{\circ }$N–26$^{\circ }$N)			
NMME:1	−0.02	−0.03	0.40
NMME:2	0.18	−0.19	0.50
Extended region: (70$^{\circ }$E–90$^{\circ }$E, 10$^{\circ }$N–30$^{\circ }$N)			
NMME:1	0.30	0.47	0.19
NMME:2	0.24	0.25	0.0

**Fig. 7:**
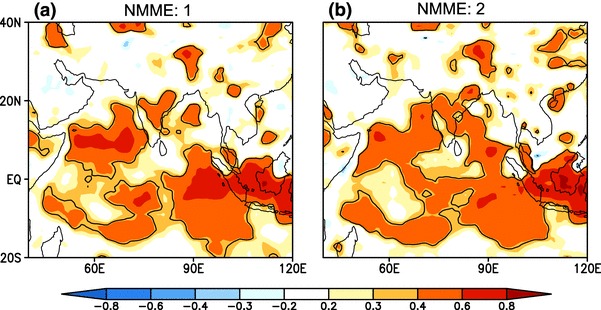
Pointwise seasonal mean rainfall anomaly correlation, **a** between multimodel ensemble mean NMME:1 and CMAP **b** between multimodel ensemble mean NMME:2 and CMAP, over the period of 1982–2009. Forecast are initialized in May and verified for JJAS mean. The contour indicates regions where correlations are statistically significant at 95%

**Fig. 8:**
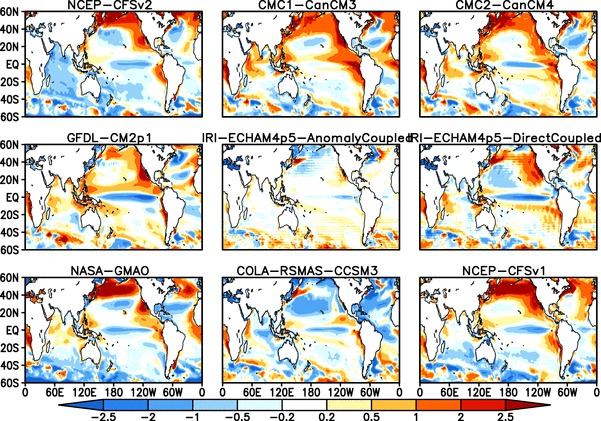
Difference between ensemble mean JJAS climatological SST of NMME:1 models and Observations (OISSTv2) (Model—Obs.), over the period of 1982–2009 , forecasts are initialized in May and verified for JJAS mean

In addition to representing climatological mean rainfall, simulation of interannual variability is another key and challenging aspect of ISMR seasonal prediction. ISMR seasonal mean rainfall shows considerable year-to-year variability, which is known to be strongly influenced by slowly varying boundary conditions (Charney and Shukla 1981). The skill of the NMME in representing the interannual variability of ISMR is shown in Fig. 6. Standardized seasonal rainfall anomalies for CMAP (red), MMEM of NMME:1 (green) and NMME:2 (Blue) are represented as histograms for each year for the CI (Fig. 1b) and extended region (Fig. 1a) for the period 1982–2009. Similar to the seasonal means, there is no significant improvement in this metric in NMME:2 relative to NMME:1. Over the CI region NMME:2 has a correlation of 0.42 with observations, while NMME:1 is 0.39. The difference is similar for the extended region (70$^{\circ }$E–90$^{\circ }$E, 10$^{\circ }$N–30$^{\circ }$N), where correlations are 0.46 for NMME:2 and 0.40 for NMME:1. To evaluate the forecast skill of seasonal mean rainfall, we have compared standardized anomalies relative to the model climatology. Thus the systematic errors are removed and standardized anomalies below or above one standard deviation are considered as drought or flood years, respectively. The MMEM of NMME:2 is able to simulate drought during 1987, 1992, 2002 and floods during 2005, while giving false alarms for droughts in 1997 and for flood in 1985 1999 and 2007 over the CI region. The NMME:2 is able to forecast normal monsoon years more skillfully as compared to extreme monsoon rainfall. Heidke skill score (Heidke 1926) for the forecast verification is calculated for the MMEM of NMME:1 and NMME:2 for the period of 1982–2009 over the CI region and the extended region (70$^{\circ }$E–90$^{\circ }$E, 10$^{\circ }$N–30$^{\circ }$N) . Standardize seasonal rainfall anomalies of CMAP, MMEM of NMME:1 and NMME:2 are divided into three categories, (above normal exceeding upper tercile), below normal (lower tercile) and normal (between both tercile) on the basis of the observed rainfall anomalies. Heidke skill scores (HSS) for both of the regions are shown in Table 2. While there is no standard cut off value for HSS for a forecast to be considered ?good?, values above 0.2 are considered as good scores. As we can see that in Table 2, HSS over central Indian region is poor for the normal and above normal seasonal mean monsoon rainfall in NMME:1 and NMME:2, while below normal monsoon seasonal mean rainfall is predicted with high HSS for both of NMME phases. Over the extended region (70$^{\circ }$E-90$^{\circ }$E, 10$^{\circ }$N-30$^{\circ }$N), HSS is moderately positive except for below normal rainfall in NMME:2 and the forecasts are skillful as compared to chance forecast in both phases of NMME. For the central India region only NMME:2 forecasts are skillful as compared to chance

Turning our attention to the spatial distribution of model skill, we show the pointwise anomaly correlation coefficient between the MMEMs of NMME:1 and NMME:2 and observations for seasonal mean rainfall (Fig. 7a, b, respectively) for the period of 1982–2009. Both phases of the NMME have higher skill over the ocean relative to land. The NMME ensembles have no skill over the northwestern part of India but show moderate skill over the central and northeastern regions. We find that skill for the NMME ensembles is improved over the Bay of Bengal, the Arabian sea, and the equatorial Indian ocean in NMME:2.

**Fig. 9:**
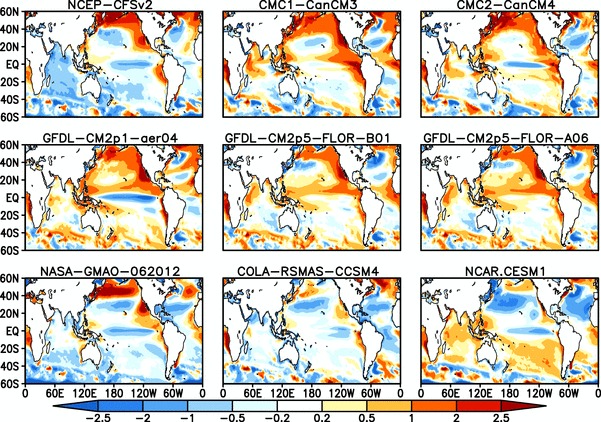
Difference between ensemble mean JJAS climatological SST of NMME:2 models and Observations (OISSTv2) (Model—Obs.), over the period of 1982–2009 , forecasts are initialized in May and verified for JJAS mean

**Fig. 10:**
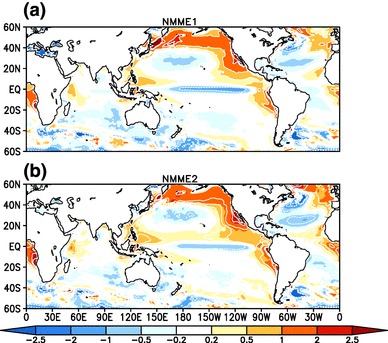
Difference between multimodel ensemble mean JJAS climatological SST from observations (OISSTv2) (Model—Obs.), **a** for NMME:1 and **b** for NMME:2, over the period of 1982–2009, forecasts are initialized in May and verified for JJAS mean

As noted in the Introduction, the interannual and intraseasonal variability of ISMR is strongly influenced by SST variability over the Pacific and Indian Oceans. Positive anomalies over the eastern Pacific (El Niño events) tend to produce below normal rainfall over India, while negative anomalies (La Niña events) lead to above normal rainfall (e.g. Sikka 1980; Pant and Parthasarathy 1981; Rasmusson and Carpenter 1983; Gadgil et al. 2003, 2004). SST over the Indian Ocean has also been shown to have a strong link with ISMR (Saji et al. 1999; Ashok et al. 2001; Krishnamurthy and Kirtman 2009). Thus, simulation of SST and the teleconnections with ISMR are important for accurate representation of the seasonal mean ISMR and its interannual variability. Differences between the ensemble mean SST bias for JJAS over the period of 1982–2009 for NMME:1 and NMME:2 are shown in Figs. 8 and 9, respectively. The NMME models are cold biased by 1–2$^{\circ }$K over the equatorial Pacific Ocean, with the exception of the GFDL-CM2p5-FLOR which has a warm bias over the same region. Northern Pacific warm biases and northern Atlantic Ocean cold biases are a common feature of all NMME models. SST over the Indian Ocean is more inconsistent, with cold biases in some models and warm biases in others. For example, CFSv2 has a large cold bias over Indian Ocean while Can-CM3, Can-CM4, GFDL-aer04 and NCAR-CESM1 show warm biases. Figure 10 shows the grand ensemble mean of JJAS mean SST between 60$^{\circ }$S–60$^{\circ }$N. The equatorial Pacific Ocean cold bias is present in both sets of hindcasts, but is improved in NMME:2 relative to NMME:1. The west Pacific is biased warm in both sets of hindcasts, but the warm bias is increased in NMME:2. The Indian Ocean MMEMs have less bias than the individual model hindcasts, due to error cancellation. The Pacific and Atlantic Ocean are both biased cold between 20$^{\circ }$N-40$^{\circ }$N by 1-2 $^{\circ }$K, and we again find the bias increases from NMME:1 to NMME:2. Overall, the seasonal mean SST biases are slightly larger in NMME:2 compared to NMME:1

**Fig. 11:**
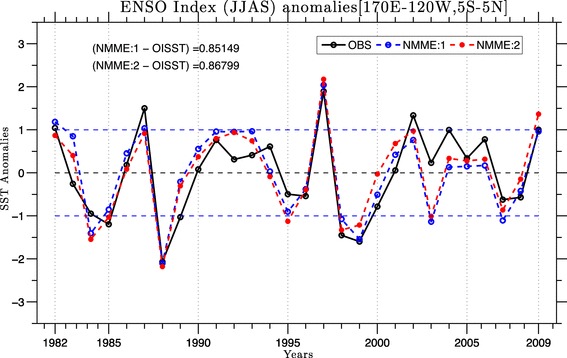
JJAS mean Nino 3.4 (area averaged SSTA (170$^{\circ }$E–120$^{\circ }$W, 5$^{\circ }$S–5$^{\circ }$N) index over the period of 1982–2009 from OISSTv2 (black solid), from MMEM of NMME:1 (dashed blue) and from MMEM of NMME:2 (dashed red), forecasts are initialized in May and verified for JJAS mean

The biases in the equatorial Pacific Ocean are particularly significant for the simulation of the monsoon, as this region is known to act as the dominant forcing in the observed interannual variability of ISMR (Kumar et al. 2006). Accurate simulation of SST anomalies over this region is thus critical to skillful dynamical seasonal prediction of the Indian monsoon (Shukla and Paolino 1983; Kumar et al. 1999; Gadgil et al. 2007). Figure 11 shows a comparison of the JJAS Niño 3.4 index (standardized area average SST average over the region 170$^{\circ }$E- 120$^{\circ }$W, 5$^{\circ }$S–5$^{\circ }$N) from OISSTv2 for NMME:1 and NMME:2 for the period of 1982–2009. We can see that the MMEMs are able to capture all major El Niño events except for 2002 and all La Niña events except for 2003 and 2007. Overall the grand means for NMME:1 and NMME:2 are able predict the seasonal mean SST anomalies with high correlation scores of 0.85 and 0.86, respectively.

**Fig. 12:**
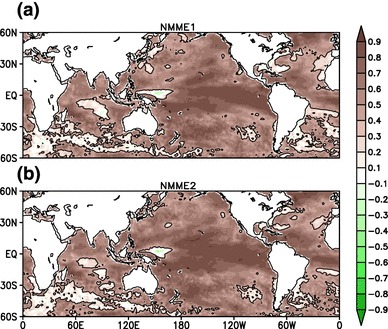
Pointwise JJAS mean SST anomaly correlation, **a** between multimodel ensemble mean NMME:1 and OISSTv2 (**b**) between multimodel ensemble mean NMME:2 and OISSTv2, over the period of 1982–2009. Forecasts are initialized in May and verified for JJAS mean. The contour indicates regions where correlations are statistically significant at 95%

In Fig. 12 we show the pointwise anomaly correlation for JJAS mean SST between the grand ensemble mean from the NMME hindcasts and observed SST over the period of 1982–2009 between 60$^{\circ }$S–60$^{\circ }$N. Correlations with observed SST are high for both of the NMME hindcasts in the central and eastern tropical Pacific Ocean, with magnitudes increasingly slightly in NMME:2. Overall skill remains greater than 0.6 everywhere except for the warm pool region in the western Pacific Ocean. SST forecast skill is improved over the Indian, Atlantic and Pacific Ocean basins in NMME:2 as compared to NMME:1.

**Fig. 13:**
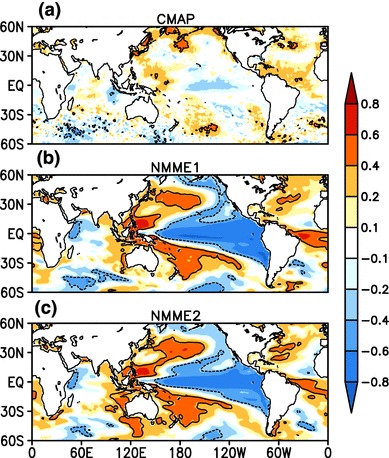
Correlation coefficient between central india (75$^{\circ }$E–85$^{\circ }$E, 16$^{\circ }$N–26$^{\circ }$N) averaged JJAS mean rainfall and JJAS mean SST between 60$^{\circ }$S–60$^{\circ }$N over the period 1982–2009 for **a** CMAP and OISSTv2, **b** for MMEM of NMME:1, **c** for MMEM of NMME:2, forecasts are initialized in May and verified for JJAS mean. The contour indicates regions where correlations are statistically significant at 95%

Some earlier studies (e.g., Palmer et al. 1992; Wang et al. 2008) suggest that long lead predictability of Indian monsoon comes from predictability of the El Niño-Southern Oscillation (ENSO). Under this theory, skillful prediction of seasonal mean rainfall and its interannual variability depends upon how skillfully models simulate ENSO variability and how well ENSO-monsoon teleconnections are represented in simulations. This motivates us to examine the simultaneous correlation between seasonal mean ISMR index over the CI region, JJAS mean SST from observations, and the grand mean of NMME:1 and NMME:2 (Fig. 13). We can see that ISMR is strongly influenced by SST in NMME hindcast as compared to observations. Rainfall over CI is negatively correlated with eastern-equatorial Pacific Ocean SST in the observations, but in the grand means of the NMME hindcasts, the rainfall-SST coupling is much stronger. We also find that SST over north Atlantic Ocean is positively correlated with the rainfall over India, similar to the simultaneous positive correlation between ISMR and subtropical Atlantic SST anomalies found by Rajeevan and Sridhar (2008). This relationship is well simulated in the grand mean of the NMME hindcasts, but it is overestimated as compared to observations. SST anomalies over the warm pool region are positively correlated with ISMR, and the association is well captured by the NMME hindcasts. In contrast, the positive simultaneous correlations between north Pacific SST anomalies and ISMR are too strong. The overestimation of the magnitude of the ENSO-monsoon teleconnection is similar to that of the DEMETER and ENSEMBLE experiments (Preethi et al. 2010; Rajeevan et al. 2012). However, in making the comparison between observations and ensemble mean one should recall that ensemble averaging suppresses the influence of climate noise (see Cash et al. 2017), which can lead to increased correlation strength relative to observations. It is important to note that SST in the Indian Ocean is also linked to interannual variability of ISMR (Saji et al. 1999; Webster et al. 1999), and that Fig. 13 indicates teleconnections between ISMR and SST in Indian Ocean are not well simulated in the NMME hindcasts. In the observations SST anomalies over the Western Equatorial Indian Ocean (WEIO: 50$^{\circ }$E–70$^{\circ }$E, 10$^{\circ }$S–10$^{\circ }$N) are positively correlated with ISMR, while they are negatively correlated in the NMME hindcasts. Likewise Eastern Equatorial Indian Ocean (EEIO: 90$^{\circ }$E–110$^{\circ }$W, 0$^{\circ }$–10$^{\circ }$S) SST anomalies are positively correlated with ISMR in NMME hindcasts, while in the observations they are negatively correlated. The NMME hindcasst thus do not capture the teleconnection between ISMR and Indian Ocean SST anomalies. This problem is by no means unique to the NMME; a study by Nanjundiah et al. (2013) analyzed retrospective forecast from 7 coupled ocean atmosphere models and found that only ECMWRF correctly simulated the Equatorial Indian Ocean Oscillation (EQUINO)-ISMR link. This may be one reason why the NMME falsely predicted droughts during the normal monsoon years of 1997 and a normal monsoon year during the flood year of 1983 (note that there is no major difference in teleconnection pattern between NMME:1 and NMME:2). SST anomalies in the Indian Ocean during 1997 and 1983 are thought to have played an important role in overcoming the negative impact of ENSO during the same years (e.g. Gadgil et al. 2007). It is interesting to note that SST in the Indian Ocean is less biased in comparison to the Pacific and Atlantic Oceans but the Indian Ocean telconnection with ISMR is represented more poorly in the NMME hindcasts. Another interesting point to be noted from Fig. 7b of Nanjundiah et al. (2013) is that even though ECMWF can capture Indian Ocean teleconnections correctly this model still gives false alarm of droughts during the normal monsoon years of 1983 and 1997.

**Fig. 14:**
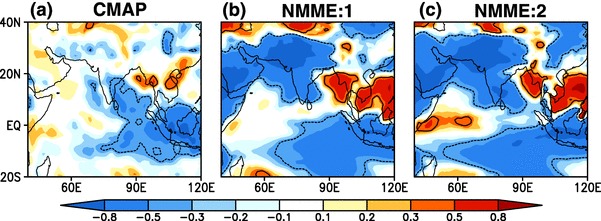
Simultaneous correlation coefficient between JJAS mean Nino 3.4 (area averaged SSTA (170$^{\circ }$E–120$^{\circ }$W ,5$^{\circ }$S–5$^{\circ }$N) index and JJAS mean rainfall anomaly over Indo–Pacific domain from 1982–2009 for **a** OISSTv2 and CMAP, **b** for MMEM of NMME:1, **c** for MMEM of NMME:2, forecasts are initialized in May and verified for JJAS mean. The contour indicates regions where correlations are statistically significant at 95%

Finally, we turn our attention to the remote association between the tropical Pacific and Indo–Pacific precipitation. As shown in Fig. 12, SST anomalies in the eastern Pacific Ocean are predicted skillfully, providing potential predictability for regional rainfall. Pointwise simultaneous correlations between the Niño 3.4 index and seasonal mean rainfall over Indo-Pacific domain are shown in Fig. 14 . Rainfall over the Indian landmass, the equatorial Indian Ocean and the maritime continent are all strongly anti-correlated with Niño 3.4 in the NMME hindcasts. NMME hindcast is thus able to simulate the correct sign of the association, but overestimates the magnitude ENSO-ISMR relationship relative to the observations. The strong ENSO-ISMR relationship suggests an overly strong oceanic influence on the atmosphere in the NMME, although as noted above the magnitude of the correlations may be exaggerated by use of the ensemble mean. However a cursory examination(results not shown here) indicates that even in time series formed by selecting individual ensemble members, the ENSO-ISMR correlation is higher than the observed.

## Conclusions

In this study we have analyzed the seasonal mean and interannual variability of the Indian summer monsoon rainfall (ISMR) in nine global ocean-atmosphere coupled models participating in the NMME:1 and nine global ocean-atmosphere coupled models participating in the NMME:2 from 1982–2009. Models are evaluated over the Indo-Pacific domain starting from May initial conditions. The two phases of the NMME are compared to each other, in part to determine what progress, if any, has been made through the inclusion of new models and improved versions of existing models in the NMME. We find the MMEM of the NMME represents seasonal mean rainfall with a modest level of skill. Both phases of the NMME simulate excessive rainfall over the western Indian Ocean and the foothills of the Himalayas, while producing deficient rainfall over the Bay of Bengal and the eastern Indian Ocean. The deficient rainfall over the Bay of Bengal and excessive rainfall over Himalayan foothills are the most common bias among the all NMME models, while biases over the Indian land region can be of either sign. Biases in the MMEMs in both phases of the NMME are strongly reflective of those of the individual models. Improvements in modeling systems and data assimilation systems is not reflected in simulations of seasonal mean of ISMR because improvements in each modeling system are small as compared to their individual mean bias.

These common systematic biases across the models likely limit the performance of the MMEM method for the seasonal prediction of ISMR. Previous studies (e.g. Zhou et al. 2009) have also shown that the performance of MMEMs is relatively low for the Indian and Asian monsoon regions. This is may be due uncertainties in the representation of subgrid scale process and coupling between large scale circulation and convection in MMEMs in general and reflected in the NMME.

The majority of rainfall over the Indian sector falls during June-September, with year to year variability of ISMR at roughly 10% of the seasonal mean. Accurate prediction of this relatively low amplitude interannual variability is a challenging and important aspect of seasonal prediction. To examine the skill of intrannual variations we have used the correlation coefficient between predicted and observed seasonal mean rainfall as a metric. As noted in the Introduction, an objective of this study is to compare the seasonal mean monsoon rainfall skill of NMME phase 1 and NMME phase 2. Earlier work (Kirtman et al. 2014) indicated that improvement in data assimilation and modeling systems contributed to improved forecast quality in NMME phase 2. However, we find the skill of seasonal prediction of Indian summer monsoon rainfall is nearly the same in NMME:2 (0.46) as compared to NMME:1 (0.40); the NMME is still not able to accurately predict extremes (drought/floods) of rainfall. Therefore seasonal monsoon rainfall forecast is not improved by the improvement in data assimilation system and modeling system in the NMME phase 2. The inability to predict extremes can also be seen in both the DEMETER and ENSEMBLE experiments (Preethi et al. 2010; Rajeevan et al. 2012). Both DEMETER and ENSEMBLE, as well as NMME predicted droughts during the normal monsoon years of 1997 and normal monsoon year during flood year of 1983. This suggests that similar biases found in the DEMETER and ENSEMBLE models exist in the models used in the NMME.

The interannual and intraseasonal time scale variability of ISMR is strongly influenced by SST variability in the Pacific and Indian Oceans. Pointwise correlation of seasonal mean SST from NMME and observations revealed that the skill of interannual predictions is high (0.6–0.9) for most ocean basins, and improved in NMME:2 relative to NMME:1. The most common seasonal mean SST biases in NMME models are cold equatorial Pacific and subtropical Atlantic Ocean and warm biases in northern Pacific Ocean. These biases also remain in the MMEMs, and while the cold bias over the equatorial Pacific is improved in NMME:2, the re-forecasts of the Indian Ocean warm bias worsen. We find that the NMME simulates the observed interannual variability of the NINO3.4 index with correlations greater than 0.8. We also find that predictions of the ENSO anomalies are remain same in both NMME:1 NMME:2.

In this work we also examine teleconnection patterns that affect the monsoon, and find that teleconnections in the MMEMs are stronger than in the observations. The MMEMs capture the ENSO-monsoon, Atlantic-monsoon and west Pacific-monsoon teleconnections correctly, but fail to correctly represent the association with the Indian Ocean. The EQUINO-ISMR relationship in particular is opposite to what is observed. The teleconnection between the ISMR and Indian Ocean SST also was not represented well in the DEMETER and ENSEMBLES models. This again suggests a common systematic error in coupled model forecasts. This error in association may be the reason why the NMME predicted droughts during the normal monsoon years of 1997 and a normal monsoon year during the flood year of 1983, as SST anomalies in the Indian Ocean during 1997 and 1983 played an important role in overcoming the negative impact of El Niño events (Gadgil et al. 2007). The NMME captures the negative correlation between ENSO and the monsoon, but the influence of ENSO on ISMR is stronger in the NMME than is observed. The overly strong ENSO-ISMR relationship suggests that oceanic influence on atmosphere may be too strong in NMME, particularly when comparing the MMEM to observations.

Overall the NNME shows modest skill in predicting Indian summer monsoon rainfall and its interannual variability. However, the NMME models show common biases in rainfall over Indian Ocean, are unable to predict the extremes in seasonal rainfall, and show only modest increases in skill from NMME:1 to NMME:2. The failure to represent the monsoon-EQUINO teleconnection in particular may be a critical limitation of the models comprising the NMME, and the association between this link and the prediction of extremes of seasonal rainfall clearly warrants further investigation.
